# A Sphingosine 1-Phosphate Gradient Is Linked to the Cerebral Recruitment of T Helper and Regulatory T Helper Cells during Acute Ischemic Stroke

**DOI:** 10.3390/ijms21176242

**Published:** 2020-08-28

**Authors:** Alexandra Lucaciu, Hannah Kuhn, Sandra Trautmann, Nerea Ferreirós, Helmuth Steinmetz, Josef Pfeilschifter, Robert Brunkhorst, Waltraud Pfeilschifter, Julien Subburayalu, Rajkumar Vutukuri

**Affiliations:** 1 Department of Neurology, Goethe University Frankfurt, 60528 Frankfurt am Main, Germany; hannah.kuhn@gmail.com (H.K.); h.steinmetz@em.uni-frankfurt.de (H.S.); rbrunkhorst@ukaachen.de (R.B.); waltraud.pfeilschifter@kgu.de (W.P.); 2 Institute of Clinical Pharmacology, Pharmazentrum Frankfurt, Goethe University Frankfurt, 60528 Frankfurt am Main, Germany; trautmann@med.uni-frankfurt.de (S.T.); ferreirosbouzas@em.uni-frankfurt.de (N.F.); 3 Institute of General Pharmacology and Toxicology, Pharmazentrum Frankfurt, Goethe University Frankfurt, 60528 Frankfurt am Main, Germany; pfeilschifter@em.uni-frankfurt.de; 4 Department of Neurology, RWTH Aachen University, 52074 Aachen, Germany; 5 Department of Medicine, University of Cambridge, Cambridge CB2 0QQ, UK; js2380@cam.ac.uk

**Keywords:** sphingosine 1-phosphate, regulatory T helper cells, stroke, sphingosine kinase, sphingosine 1-phosphate receptor, ceramides, fingolimod

## Abstract

Emerging evidence suggests a complex relationship between sphingosine 1-phosphate (S1P) signaling and stroke. Here, we show the kinetics of S1P in the acute phase of ischemic stroke and highlight accompanying changes in immune cells and S1P receptors (S1P_R_). Using a C57BL/6 mouse model of middle cerebral artery occlusion (MCAO), we assessed S1P concentrations in the brain, plasma, and spleen. We found a steep S1P gradient from the spleen towards the brain. Results obtained by qPCR suggested that cells expressing the S1P_R_ type 1 (S1P_1_^+^) were the predominant population deserting the spleen. Here, we report the cerebral recruitment of T helper (T_H_) and regulatory T (T_REG_) cells to the ipsilateral hemisphere, which was associated with differential regulation of cerebral S1P_R_ expression patterns in the brain after MCAO. This study provides insight that the S1P-S1P_R_ axis facilitates splenic T cell egress and is linked to the cerebral recruitment of S1P_R_^+^ T_H_ and T_REG_ cells. Further insights by which means the S1P-S1P_R_-axis orchestrates neuronal positioning may offer new therapeutic perspectives after ischemic stroke.

## 1. Introduction

In the last two decades, the formerly enigmatic sphingolipids and their metabolism have aroused a lot of biomedical research interest due to their pivotal role as signaling molecules. The regulation of a wide range of cellular processes was described, including endocytosis, intracellular trafficking of molecular constituents, and signal transduction by membrane receptors [1,2]. Recent insights into the molecular mechanisms of action provide increasing evidence for the complex pathways of sphingolipid metabolism in the pathogenesis of multiple diseases including cancer, diabetes, neurodegenerative disorders, autoimmune diseases, and stroke [3,4,5,6,7]. Fingolimod represents an unselective functional antagonist to sphingosine 1-phosphate receptors (S1P_R_) resulting in S1P_R_ downregulation upon S1P_R_ engagement [8,9]. The successful application of fingolimod in the treatment of various diseases characterized by a perturbed S1P metabolism suggests the strong therapeutic potential of the S1P-S1P_R_-axis [8,10,11,12].

To date, S1P is conceived to regulate immune cell recruitment towards high S1P gradients [13,14] by ligating the five bona fide S1P_R_ type 1–5 (S1P_1–5_) [15]. This gradient is perturbed under diseased conditions and S1P_R_ signaling acts as a driver of multiple diseases [16]. However, how the S1P-S1P_R_-axis contributes to pathology in focal cerebral ischemia has not been elucidated in all details [17,18,19].

Ischemic stroke holds a high mortality [20] and its treatment is currently limited to restoring the impaired blood flow [21]. Moreover, neuroprotectants have failed to add on currently available therapeutic options [22]. In contrast, characterizing the dysfunction and elucidating the mechanism of recovery in the peri-infarct cortex, i.e., the non-ischemic tissue surrounding the ischemic core, may possess therapeutic potential and protect from delayed secondary damage after ischemic stroke [23]. In that respect, fingolimod has been shown to improve outcome after stroke in preclinical and clinical trials [5,6,24,25].

The first well-characterized biological effects of S1P signaling were described in immune cells [26], demonstrating a key role of S1P and S1P_R_ ligation in regulating T cell distribution [9,14]. In stroke research, evidence has accumulated that focal cerebral ischemia facilitates an acute inflammatory cascade characterized by the infiltration of inflammatory cells and, in particular, T cells in an early and antigen-independent manner into the ischemic brain to contribute to post-ischemic brain damage [27,28,29]. The detrimental impact of lymphocyte infiltration in early ischemic brain injury led by T and B lymphocytes as injurious players was shown in experimental stroke by T and B cell-deficient mice (SCID) [30], as well as asplenic rats [31]. In addition, studies suggest a dynamical reflection in the peripheral immune system [32], leading to a state of immunosuppression. A better characterization of the migration of immune cells into the ischemic cerebral parenchyma represents a prerequisite for therapeutic interventions [33].

Therefore, the aims of this study were to unravel the kinetics of the S1P metabolism and the differential regulation of S1P_R_ on systemic alterations of immune cell populations with reference to their cerebral recruitment in the acute phase of ischemic stroke. Understanding the mechanisms by which immune cell subsets are recruited to the brain via S1P could widen therapeutic options in ischemic stroke.

## 2. Results

### 2.1. Acute Ischemic Stroke Leads to Increased Plasma S1P Levels and Creates a Gradient between the Ischemic Core and the Peri-Infarct Cortex

**Figure 1:**
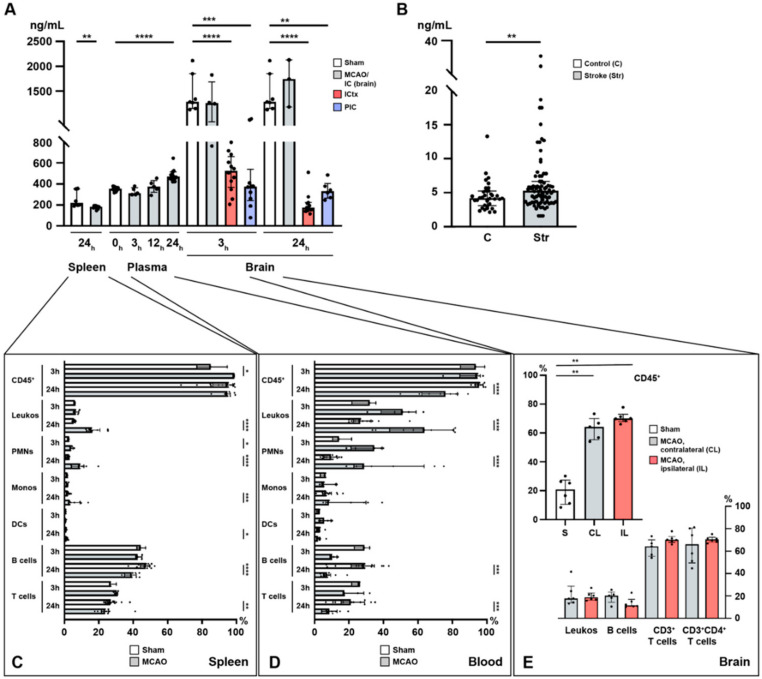
Temporal profile of immuno-cellular and sphingosine 1-phosphate (S1P) alterations after acute ischemic stroke in the spleen, circulation, and the brain. (**A**) Murine S1P levels are shown in the spleen (24 h), the plasma (3, 12, and 24 h), and the brain (3 and 24 h) after MCAO. (**B**) Enhanced S1P plasma levels in patients with ischemic stroke. (**C**) Alterations in immune cell populations in the murine spleen (3 and 24 h) after middle cerebral artery occlusion (MCAO). (**D**) Alterations in immune cell populations in the murine blood (3 and 24 h) after MCAO. (**E**) CD45^+^ pan-immune cells are expanded in the murine brain 24 h after MCAO. The vast majority of these CD45^+^ cells comprised T cells. IC: ischemic core; ICtx: ischemic cortex; PIC: peri-infarct cortex. The Mann–Whitney U-test was applied to calculate statistical differences, except for the mouse plasma data (Kruskal–Wallis test) and for the human stroke samples (unpaired t test with Welch’s correction). The data are presented as median ± IQR; * *p* < 0.05, ** *p* < 0.01, *** *p* < 0.001, **** *p* < 0.0001.

In order to study the potential of S1P to act as a chemotactic agent in cerebral ischemia, we established a temporal profile of S1P concentrations in the murine spleen, plasma, and brain in a model of acute cerebral ischemia (MCAO). Under homeostasis, a steep S1P gradient exists towards the brain with the lowest concentration found in the spleen and a moderate concentration observed in the plasma (Figure 1A). In the plasma, a significant S1P increase was observed 24 h after stroke (sham vs. MCAO; 352.8 ± 20.0 ng/mL vs. 482.4 ± 55.0 ng/mL, *p* < 0.0001, Figure 1A). Prior to 24 h, no significant change could be reported both at 3 h (sham vs. MCAO; 352.8 ± 20.0 ng/mL vs. 327.5 ± 35.9 ng/mL, *p* = 0.168, Figure 1A) and 12 h after MCAO (sham vs. MCAO; 352.8 ± 20.0 ng/mL vs. 375.6 ± 61.3 ng/mL, *p* = 0.3056, Figure 1A). In humans, who had suffered from acute ischemic stroke (Str), we were able to measure similar changes 24 h after onset of ischemia as compared to healthy controls (C) (C vs. Str; 4.55 ± 2.1 ng/mg vs. 6.53 ± 5.0 ng/mg, *p* = 0.0034, Figure 1B).

In the brain, S1P remains high in the ischemic core (IC) with a tendency to further increase within the first 24 h, although statistical significance could not be reached due to a small sample size (3 h: sham vs. MCAO; 1448 ± 380.4 pg/mg vs. 1275 ± 435.4 pg/mg, *p* = 0.4121, Figure 1A; 24 h: sham vs. MCAO; 1448 ± 380.4 pg/mg vs. 1685 ± 477.8 pg/mg, *p* = 0.5167, Figure 1A; IC_3h_ vs. IC_24h_; 1275.0 ± 435.4 vs. 1685.0 ± 477.8, *p* = 0.6286, Figure 1A). In contrast, S1P was reduced 3 h after the MCAO challenge had occurred in the peri-infarct cortex (PIC) (sham vs. MCAO; 1448 ± 380.4 pg/mg vs. 425.0 ± 284.8 pg/mg, *p* = 0.0001, Figure 1A), as well as in the ischemic cortex (ICtx) (sham vs. MCAO; 1448 ± 380.4 pg/mg vs. 509.0 ± 180.4 pg/mg, *p* < 0.0001, Figure 1A) compared to the corresponding cortex in sham-operated mice. The reduction in S1P levels in the PIC persisted over 24 h (sham vs. MCAO; 1448 ± 380.4 pg/mg vs. 340.6 ± 85.2 pg/mg, *p* = 0.0012, Figure 1A) but was unchanged compared to 3 h after MCAO (PIC_3h_ vs. PIC_24h_; 425.0 ± 284.8 vs. 340.6 ± 85.2, *p* = 0.6159, Figure 1A). This reduction allowed a new S1P gradient to be established between the IC and the PIC (3 h: IC vs. PIC; 1275.0 ± 435.4 vs. 425.0 ± 284.8, *p* = 0.007, Figure 1A), which even steepened at 24 h (24 h: IC vs. PIC; 1685.0 ± 477.8 vs. 340.6 ± 85.2, *p* = 0.0238, Figure 1A). The decrease in the ICtx also prevailed at 24 h (sham vs. MCAO; 1448 ± 380.4 pg/mg vs. 210.8 ± 109.2 pg/mg, *p* < 0.0001, Figure 1A) and consolidated (ICtx_3h_ vs. ICtx_24h_; 509.0 ± 180.4 vs. 210.8 ± 109.2, *p* = 0.0002, Figure 1A).

In the spleen, S1P was reduced 24 h after MCAO (sham vs. MCAO; 256.5 ± 77.9 pg/mg vs. 178.5 ± 16.0, *p* = 0.0027, Figure 1A).

### 2.2. Lymphocyte Evasion from the Spleen after Acute Ischemic Stroke

Next, we assessed the immune cell traffic between these compartments in the context of stroke. Our data suggest an evasion of splenic lymphocytes (T and B cells) and a striking regulation of all leukocyte populations in the circulation (blood). As an established surrogate marker for immune cell egress, the spleen weight was evaluated after MCAO (Figure S1) yielding a relevant reduction in splenic weight as early as 12 h after MCAO (sham vs. MCAO; 70.0 ± 12.3 mg vs. 50.0 ± 7.1 mg, *p* = 0.0238, Figure S1). This reduction was even more pronounced 24 h after MCAO (sham vs. MCAO; 80.0 ± 19.1 mg vs. 48.3 ± 8.3 mg, *p* < 0.0001, Figure S1).

Flow cytometric analysis of spleen homogenates revealed relevant changes in terms of decreasing relative cell numbers in B and T cells, although this change took 24 h in our study to reach statistical significance. Here, B cell frequencies remained unchanged at 3 h (sham vs. MCAO; 44.6 ± 2.5 vs. 43.1 ± 1.9, *p* = 0.57, Figure 1C) and decreased at 24 h (sham vs. MCAO; 46.6 ± 3.9 vs. 39.0 ± 3.5, *p* < 0.0001, Figure 1C). Similarly, at 3 h no change was observed in T cells (sham vs. MCAO; 27.9 ± 2.0 vs. 30.1 ± 1.1, *p* = 0.1429, Figure 1C), whilst a reduction was detected at 24 h (sham vs. MCAO; 27.7 ± 3.8 vs. 24.7 ± 6.1, *p* = 0.0093, Figure 1C). Additionally, we measured the pro-inflammatory IL-6 (a pleiotropic cytokine), and the transcription factors (TFs) SPI1 (B cell TF), STAT3 (T_H_ cells), and FoxP3 (T_REG_ cells) (Figure S2). Indeed, we found severe alterations in the expression level of IL-6 at 3 and 24 h after MCAO suggesting pan-immune cell egress (3 h: sham vs. MCAO; 1.0 ± 0.4 vs. 0.0 ± 0.0, *p* = 0.0005; 24 h: sham vs. MCAO: 1.0 ± 0.4 vs. 0.2 ± 0.1, *p* < 0.0001, Figure S2). In terms of specific cell populations, we measured SPI1, which suggested early B cell egress from the spleen at 3 h (sham vs. MCAO; 1.0 ± 0.2 vs. 0.3 ± 0.1, *p* = 0.001, Figure S2). In contrast, these effects were revoked as indicated by levels of SPI1 at 24 h comparable to sham (sham vs. MCAO; 1.0 ± 0.2 vs. 1.1 ± 0.3, *p* = 0.2775, Figure S2). Unlike B cells, T_H_ and T_REG_ cells remained reduced at 3 and 24 h alike, although not statistically significant for T_REG_ cells (T_H_ cells identified by STAT3: 3 h: sham vs. MCAO; 1.0 ± 0.3 vs. 0.2 ± 0.0, *p* = 0.001; 24 h: sham vs. MCAO; 1.0 ± 0.3 vs. 0.4 ± 0.2, *p* = 0.0003; T_REG_ cells identified by FoxP3: 3 h: sham vs. MCAO; 1.0 ± 0.1 vs. 0.2 ± 0.0, *p* = 0.0005; 24 h: sham vs. MCAO; 1.0 ± 0.1 vs. 0.8 ± 0.8, *p* = 0.0652, Figure S2). Taken together, this hints at reduced numbers of splenic lymphocytes, in accordance with previous reports [32].

By contrast, CD45, which was used as a pan-leukocyte and pan-lymphocyte marker, showed an early increase after 3 h (sham vs. MCAO; 85.6 ± 8.8 vs. 98.6 ± 0.2, *p* = 0.036, Figure 1C). This effect was no longer seen after 24 h (sham vs. MCAO; 91.3 ± 8.7 vs. 94.8 ± 3.7, *p* = 0.5197, Figure 1C). The relative frequencies of leukocytes (CD45^+^CD11b^+^ cells) were unaffected at 3 h (sham vs. MCAO; 6.0 ± 0.4 vs. 7.0 ± 1.4, *p* = 0.25, Figure 1C) and increased after 24 h (sham vs. MCAO; 5.7 ± 0.7 vs. 17.3 ± 4.6, *p* < 0.0001, Figure 1C). Polymorphonuclear neutrophils (PMNs) characterized by the expression of Ly6G in CD45^+^ cells were increased both at 3 h (sham vs. MCAO; 2.6 ± 0.1 vs. 4.1 ± 1.1, *p* = 0.0357, Figure 1C) and 24 h after MCAO (sham vs. MCAO; 2.1 ± 0.8 vs. 9.2 ± 4.6, *p* < 0.0001). Monocyte frequencies (CD45^+^Ly6C^MODERATE^) were only increased after 24 h (3 h: sham vs. MCAO; 1.7 ± 0.2 vs. 1.8 ± 0.5, *p* = 0.7857; 24 h: sham vs. MCAO; 2.1 ± 0.8 vs. 5.3 vs. 4.0, *p* = 0.0008, Figure 1C). Dendritic cells (CD45^+^Ly6C^HIGH^) displayed only a subtle change in their frequencies (3 h: sham vs. MCAO; 0.9 ± 0.1 vs. 0.7 ± 0.4, *p* = 0.25; 24 h: sham vs. MCAO; 0.8 ± 0.3 vs. 1.4 ± 0.6, *p* = 0.0133, Figure 1C).

### 2.3. Immune Cell Alterations in the Circulation after Acute Ischemic Stroke

As in the spleen, significant alterations of immune cell counts could be appreciated in the circulation (Figure 1D). CD45^+^ cells were reduced in the circulation after 24 h (sham vs. MCAO; 95.5 ± 2.5 vs. 72.9 ± 13.1, *p* < 0.0001), but not at 3 h after MCAO (sham vs. MCAO; 92.3 ± 7.1 vs. 91.1 ± 9.3, *p* > 0.9999, Figure 1D). While the relative numbers of leukocytes increased in the circulation although this effect only reached statistical significance after 24 h (3 h: sham vs. MCAO; 29.8 ± 7.2 vs. 49.3 ± 12.0, *p* = 0.1429; 24 h: sham vs. MCAO; 29.3 ± 9.4 vs. 61.5 ± 18.6, *p* < 0.0001, Figure 1D) and this increase was reflected in neutrophil frequencies, in spite of more pronounced variation compared to the entirety of leukocytes (3 h: sham vs. MCAO; 15.5 ± 5.5 vs. 31.2 ± 9.5, *p* = 0.0714; 24 h: sham vs. MCAO; 9.4 ± 4.0 vs. 39.0 ± 22.2, *p* < 0.0001, Figure 1D), monocytes and dendritic cells remained entirely unaltered (monocytes: 3 h: sham vs. MCAO; 5.5 ± 1.8 vs. 7.9 ± 4.5, *p* = 0.9643; 24 h: sham vs. MCAO; 7.7 ± 4.1 vs. 17.2 ± 13.1, *p* = 0.0622; dendritic cells: 3 h: sham vs. MCAO; 2.3 ± 1.0 vs. 6.1 ± 3.6, *p* = 0.2286; 24 h: sham vs. MCAO; 2.6 ± 1.3 vs. 2.6 ± 1.8, *p* = 0.7564, Figure 1D). Analogous to decreased frequencies observed in the spleen, B and T cells were also reduced in the circulation after 24 h (B cells: 3 h: sham vs. MCAO; 28.1 ± 4.5 vs. 10.8 ± 2.2, *p* = 0.0571; 24 h: sham vs. MCAO; 27.1 ± 11.4 vs. 8.2 ± 4.6, *p* = 0.0004; T cells: 3 h: sham vs. MCAO; 24.7 ± 2.7 vs. 21.7 ± 6.7, *p* = 0.3929; 24 h: sham vs. MCAO; 21.7 ± 8.4 vs. 9.4 ± 5.3, *p* = 0.0007, Figure 1D).

### 2.4. Sphingosine 1-Phosphate Is Linked to Cerebral T Cell Recruitment after Acute Ischemic Stroke

Given the S1P gradient found and the decreased frequency of T_H_ and T_REG_ cells both in the spleen and circulation alike, we assumed these T cell populations would be subject to a swift recruitment towards the high S1P concentration present in the brain, which was particularly high in the IC after stroke. The release and migration of peripheral immune cells into the central nervous system (CNS) with subsequent infiltration into the ischemic brain parenchyma are conceived as a putative mechanism to potentiate brain damage [34,35]. We found that CD45^+^ immune cells were recruited to the brain 24 h after MCAO as indicated by a significant increase of CD45^+^ immune cell frequencies (Figure 1E). Here, a recruitment of CD45^+^ cells was observed to the contralateral hemisphere (CL) (sham vs. CL; 19.6 ± 8.8 vs. 63.0 ± 7.8, *p* = 0.0043, Figure 1E) and to the ipsilateral hemisphere (IL) (sham vs. IL; 19.6 ± 8.8 vs. 70.6 ± 4.0, *p* = 0.0022, Figure 1E), although no difference between the CL and the IL could be established (CL vs. IL; 63.0 ± 7.8 vs. 70.6 ± 4.0, *p* = 0.1875, Figure 1E). Of interest, the vast majority of these infiltrating immune cells comprised T_H_ cells (CD3^+^: CL vs. IL; 63.0 ± 7.8 vs. 70.6 ± 4.0; CD3^+^CD4^+^: CL vs. IL; 64.9 ± 15.7 vs. 70.4 ± 2.9, Figure 1E). In contrast, non-lymphocytic leukocytes and B cells contributed only scarcely to the CD45^+^ immune cell recruitment observed (leukocytes: CL vs. IL; 21.4 ± 10.6 vs. 19.7 ± 4.2; B cells: CL vs. IL; 19.1 ± 5.5 vs. 13.7 ± 4.8, Figure 1E).

In appreciation of these findings and in light of the S1P gradient measured we investigated the kinetics of S1P_R_ expression in the spleen and brain after acute ischemic stroke.

### 2.5. S1P_1_ Might Contribute to the Lymphocyte Evasion from the Spleen after Acute Ischemic Stroke

**Figure 2:**
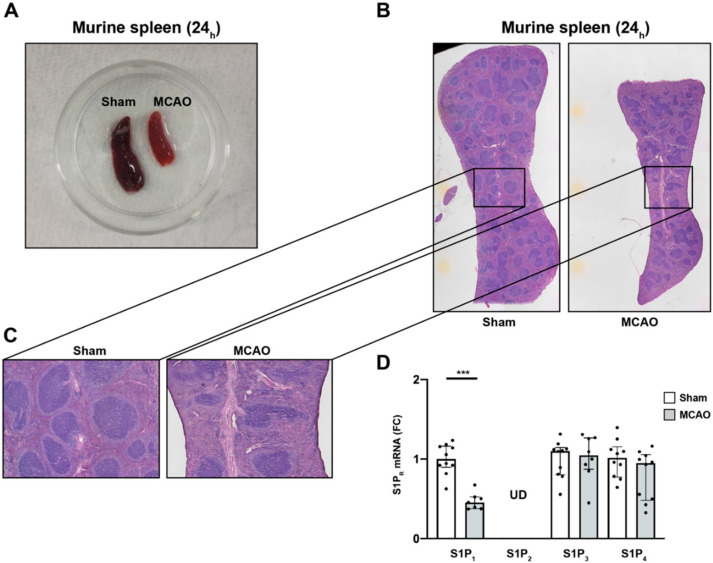
S1P_1_ immune cell egress from the spleen after acute ischemic stroke. (**A**) Macroscopic reduction in spleen size 24 h after MCAO (sham-(left) vs. MCAO-operated mice (right)). (**B**) Microscopic comparison of sham-(left) vs. MCAO-treated mice (right). (**C**) Inserts display a predominant reduction of white pulp tissue compared to the red pulp, which was only mildly affected. (**D**) Detection of S1P_1_, S1P_2_, S1P_3_, and S1P_4_ mRNA levels in the murine spleen 24 h after MCAO. S1P_2_ remained at undeterminable levels (UD) across all eight biological replicates tested. Fold change (FC) was normalized compared to splenic GAPDH mRNA levels. The Mann–Whitney U-test was applied to calculate statistical differences. Data are presented as median ± IQR; *** *p* < 0.001.

To evaluate the putative mechanism behind the chemotaxis of immune cells in light of the increasing S1P plasma levels after stroke, the relationship of S1P_R_ expression between immune cells deserting the spleen and the central nervous system (CNS) was analyzed. In this regard, it was previously postulated that splenic responses after stroke and their contribution to ischemic brain damage represent important players in the pathology underlying stroke [36]. Our findings that the spleen is subject to drastic shrinkage during the acute phase after MCAO (Figure 2A), are in accordance with previous reports [31,32]. In addition, histopathology of spleens 24 h after MCAO displayed a predominant reduction of the lymphoid tissue, in particular, and to some subtler extent hematopoietic elements in the red pulp (Figure 2B,C). Previous studies have highlighted the importance of S1P receptor expression by various immune cells, and T and B cells in particular, for lymphocyte egress from tissues [37]. Hence, we measured the S1P_R_ mRNA expression after MCAO from splenic tissue lysates. We detected a significant reduction in the mRNA levels of S1P_1_ 24 h after MCAO (sham vs. MCAO; 1.00 ± 0.2 vs. 0.5 ± 0.1, *p* = 0.0002, Figure 2D). To test S1P_1_-specificity of this phenomenon, we measured mRNA levels of S1P_2_, S1P_3_, and S1P_4_ and did not find any significant alterations (S1P_2_: undetermined (UD) in sham vs. MCAO; S1P_3_: sham vs. MCAO; 1.0 ± 0.2 vs. 1.0 ± 0.3, *p* = 0.8968; S1P_4_: sham vs. MCAO; 1.0 ± 0.2 vs. 0.8 ± 0.3, *p* = 0.1903, Figure 2D). These findings suggest predominantly S1P_1_^+^-immune cells to evade the spleen in response to the S1P gradient towards the brain secondary to an additional trigger set off by cerebral ischemia. S1P_1_ is conceived to play an important role in T cell egress from secondary lymphoid organs towards high S1P gradients [38,39]. Given that only B cells and T cells were present at lower frequencies in the spleen after ischemic stroke (Figure 1C), we assume that the expression of S1P_1_ may be required for their evasion from the spleen.

### 2.6. Recruitment of T_H_ and T_REG_ Cells to the Peri-Infarct Cortex after Stroke Is Associated with an Altered Cerebral S1P_R_ Pattern

**Figure 3:**
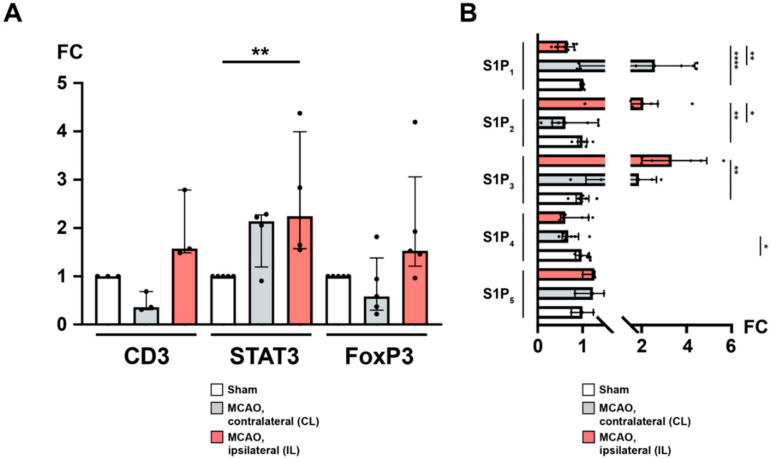
The ipsilateral hemisphere is distinguished by CD3^+^ FoxP3^+^ T_H_ cell recruitment and differential regulation of S1P receptors after acute ischemic stroke. (**A**) mRNA levels of CD3, STAT3, and FoxP3 were quantified by measuring fold change (FC) normalized to GAPDH expression in the murine brain (24 h) after MCAO. An expansion of CD3^+^ regulatory T_H_ cells was observed in the ipsilateral hemisphere. (**B**) Likewise, mRNA levels of the sphingosine 1-phosphate receptors S1P_1_, S1P_2_, S1P_3_, S1P_4_, and S1P_5_, respectively, were quantified. In particular, S1P_1_ was subject to a downregulation as opposed to upregulated S1P_2_ and S1P_3_ in the ipsilateral hemisphere. The Mann–Whitney U-test was applied to calculate statistical differences between sham (S) and the contralateral hemisphere (CL) or ipsilateral hemisphere (IL) in MCAO-treated mice. A Wilcoxon test was used when comparing CL with IL. In (**A**), each data point refers to the average of four homogenized whole brain lysates (sham), or half-brain lysates, i.e., ipsilateral or contralateral, respectively. The data are presented as median ± IQR; * *p* < 0.05, ** *p* < 0.01, **** *p* < 0.0001.

We wanted to further investigate the previously observed T cell recruitment to the brain (Figure 1E). By qPCR and quantitative immunohistochemistry (IHC), we found that the CL featured unaltered yields of mRNA for CD3 (sham vs. CL; 1.0 ± 0.0 vs. 0.5 ± 0.2, *p* = 0.1, Figure 3A), FoxP3 (sham vs. CL; 1.0 ± 0.0 vs. 0.8 ± 0.6, *p* = 0.127, Figure 3A), and STAT3 (sham vs. CL; 1.0 ± 0.0 vs. 1.9 ± 0.6, *p* = 0.2063, Figure 3A). In contrast, while CD3 and FoxP3 mRNAs were also unaffected in the IL 24 h after MCAO (CD3: sham vs. IL; 1.0 ± 0.0 vs. 2.0 ± 0.7, *p* = 0.1; FoxP3: sham vs. IL; 1.0 ± 0.0 vs. 2.0 ± 1.3, *p* = 0.127, Figure 3A), STAT3 was significantly upregulated compared to sham (sham vs. IL; 1.0 ± 0.0 vs. 2.6 ± 1.3, *p* = 0.0079, Figure 3A). Interestingly, the proclivity for enhanced FoxP3 expression in the IL was close to significance when compared to the CL (CL vs. IL; 0.8 ± 0.6 vs. 2.0 ± 1.3, *p* = 0.0625, Figure 3A).

Previously, sphingosine 1-phosphate receptors were reported to be expressed both on neuronal cells as well as on infiltrating T cells [19,40]. Hence, we were also interested in the differential regulation of sphingosine 1-phosphate receptors in response to focal cerebral ischemia. S1P_1_ was found to be downregulated in the IL both compared to sham (S1P_1_: sham vs. IL; 1.0 ± 0.0 vs. 0.6 ± 0.2, *p* < 0.0001, Figure 3B) and to the CL (S1P_1_: CL vs. IL; 2.7 ± 1.6 vs. 0.6 ± 0.2, *p* = 0.0078) suggesting an S1P-S1P_1_ ligation with subsequent receptor downregulation. There was no difference between sham and the CL (S1P_1_: sham vs. CL; 1.0 ± 0.0 vs. 2.7 ± 1.6, *p* = 0.2183, Figure 3B). Regarding S1P_2_, we could demonstrate a significant upregulation in the IL after MCAO compared to sham (S1P_2_: sham vs. IL; 1.0 ± 0.1 vs. 2.2 ± 1.1, *p* = 0.0022, Figure 3B) and the CL (S1P_2_: CL vs. IL; 0.8 ± 0.5 vs. 2.2 ± 1.1, *p* = 0.0156, Figure 3B). There was no difference between sham and the CL (S1P_2_: sham vs. CL; 1.0 ± 0.1 vs. 0.8 ± 0.5, *p* = 0.6126, Figure 3B). Similarly, S1P_3_ displayed a profound upregulation in the IL (S1P_3_: sham vs. IL; 1.0 ± 0.2 vs. 3.5 ± 1.5, *p* = 0.0022, Figure 3B; CL vs. IL; 1.9 ± 0.8 vs. 3.5 ± 1.5, *p* = 0.1875, Figure 3B). The S1P_3_ expression in the CL was not statistically different from sham (S1P_3_: sham vs. CL; 1.0 ± 0.2 vs. 1.9 ± 0.8, *p* = 0.0823, Figure 3B). Moreover, S1P_4_ and S1P_5_ were also detected in the mouse brain. Compared to sham, neither the CL nor the IL showed any difference after MCAO (S1P_4_: sham vs. IL; 1.0 ± 0.1 vs. 0.8 ± 0.3, *p* = 0.2523; CL vs. IL; 0.7 ± 0.2 vs. 0.8 ± 0.3, *p* = 0.3125; S1P_5_: sham vs. CL; 1.0 ± 0.2 vs. 1.2 ± 0.3, *p* = 0.7; sham vs. IL; 1.0 ± 0.2 vs. 1.2 ± 0.2, *p* = 0.2; CL vs. IL; 1.2 ± 0.3 vs. 1.2 ± 0.2, *p* > 0.9999, Figure 3B) except for S1P_4_, which was downregulated in the CL (sham vs. CL; 1.0 ± 0.1 vs. 0.7 ± 0.2, *p* = 0.036, Figure 3B).

Given the indication for relevant T_H_ and T_REG_ cell infiltration and a differential expression of S1P_R_ by qPCR, we sought to confirm these observations using quantitative immunohistochemistry (IHC) for CD3 and FoxP3, respectively.

We detected a significant egress of CD3 T cells from the spleen swiftly after the MCAO intervention had occurred (Figure S3). Our measurements were acquired 3 and 24 h after MCAO, respectively, but some reports do suggest that this process almost coincides with the onset of cerebral ischemia [40]. One day after the intervention, T cells re-appeared both in the white and red pulp, respectively, although at a lower expression level (Figure S3).

**Figure 4:**
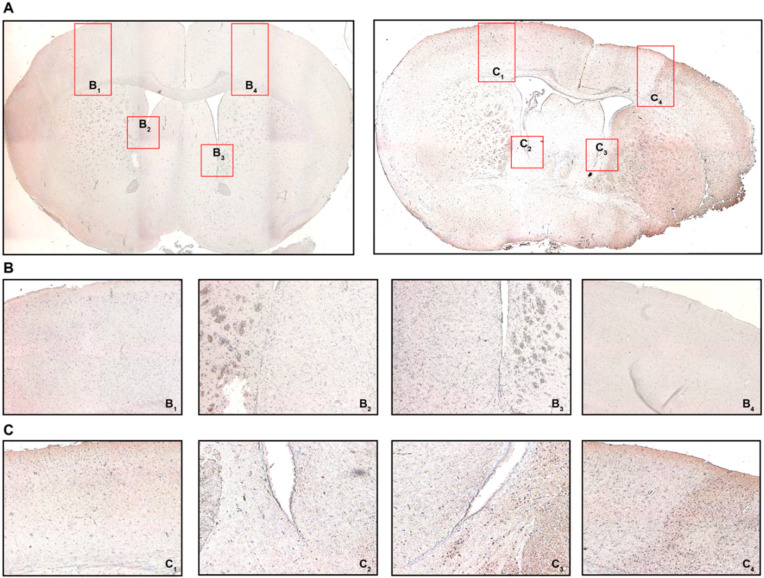
Cerebral recruitment of CD3^+^ T cells in the peri-infarct cortex and ipsilateral white matter after acute ischemic stroke. (**A**) Coronal brain sections from a sham-operated mouse (left) or a mouse subjected to MCAO (right) 24 h post-intervention. (**B**) B_1_ refers to the contralateral cortical area, B_2_ refers to the contralateral white matter adjacent to the lateral ventricle, B_3/4_ refer to the ipsilateral equivalents to B_1/2_. (**C**) C_1_ refers to the contralateral cortical area, C_2_ refers to the contralateral white matter adjacent to the lateral ventricle, C_3/4_ refer to the ipsilateral equivalents to C_1/2_. Positive CD3 immunostaining was characterized by brown-colored cells with condensed nuclei and a subtle surrounding cytoplasmic area pertinent to lymphocytes, particularly in the peri-infarct cortex (C_4_) and the ipsilateral white matter (C_3_), which was not observed in sham-operated mice (B_3_ and B_4_, respectively). Original magnifications: (**A**) 2×; (**B**,**C**) 8×.

Appreciating the reduced T cell frequencies both in the spleen and circulation 24 h after MCAO, we intended to confirm if T cells were recruited towards the brain. For this reason, we defined four regions of interest as potential areas for T cell recruitment: areas equivalent to the peri-infarct cortex, but similarly the white matter area adjacent to the ischemic area from the ipsilateral (IL) and contralateral (CL) hemisphere, respectively (Figure 4A). We found CD3 immunostaining particularly prevalent in the peri-infarct cortex (PIC) (Figure 4(C_4_)) and the associated peri-ventricular area (PVA) in the ipsilateral hemisphere (Figure 4(C_3_)) in MCAO-operated mice 24 h post-intervention. These changes, to that extent, were not observed neither in the contralateral hemisphere in MCAO mice (Figure 4(C_2_,C_1_), respectively), nor did any brain area in sham-treated mice express CD3 more abundantly (Figure 4(B_1–4_)). These changes in CD3 expression were also quantified (Figure S4).

**Figure 5:**
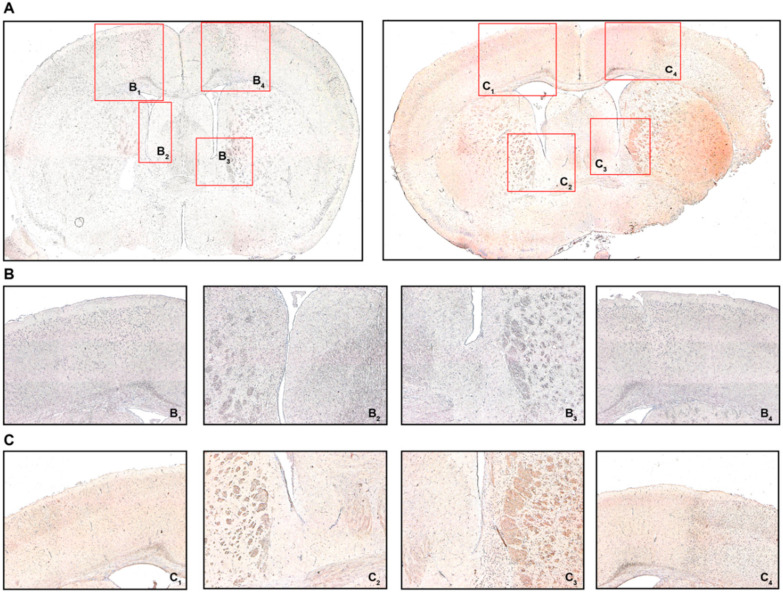
FoxP3 immunohistochemistry (IHC). (**A**) Coronal brain sections from sham-operated mice (left) or MCAO-mice (right) 24 h post-intervention detailing where the respective detailed images were taken from. (**B**) B_1_ refers to the contralateral cortical area, B_2_ refers to the contralateral white matter adjacent to the lateral ventricle, B_3/4_ refer to the ipsilateral equivalents to B_1/2_. (**C**) C_1_ refers to the contralateral cortical area, C_2_ refers to the contralateral white matter adjacent to the lateral ventricle, C_3/4_ refer to the ipsilateral equivalents to C_1/2_. Positive FoxP3 immunostaining was characterized by brown-colored cells with condensed nuclei and a subtle surrounding cytoplasmic area pertinent to lymphocytes, particularly in the peri-infarct cortex (C_4_) and the ipsilateral white matter (C_3_), which was not observed in sham-operated mice (B_3_ and B_4_, respectively). Original magnifications: (**A**) 2×; (**B**,**C**) 8×.

Using IHC we also found FoxP3 to be expressed by some of the infiltrating cells (Figure 5). Similar to CD3, only the ipsilateral PIC and PVA in MCAO mice did demonstrate relevant FoxP3 immunostaining compared to the CL and sham-operated mice (Figure 5A–C). Quantification revealed very low levels of FoxP3 positivity (Figure S5), aligning well with the literature [41].

### 2.7. Ceramide Species: A Conflicting Chemotactic Agent for Immune Cell Egress?

**Figure 6:**
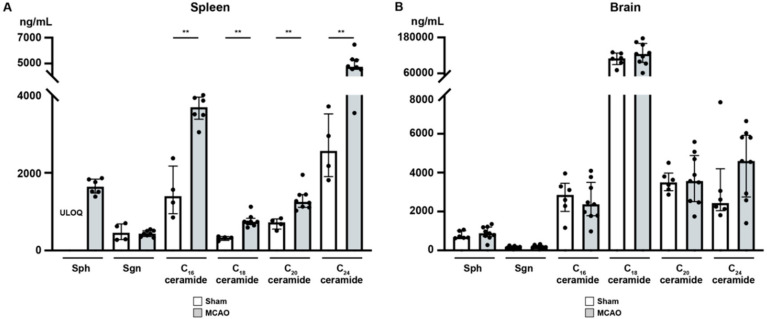
Sphingosine, sphinganine, and ceramide species levels in the spleen and murine brain (24 h) after MCAO acute ischemic stroke. (**A**) Across all the ceramide species tested an increase was seen in the murine spleen (24 h) after MCAO. (**B**) Ceramide species levels in the peri-infarct cortex were unaffected (24 h) after acute ischemic stroke. The Mann–Whitney U-test was applied to calculate statistical differences. The data are presented as median ± IQR; ** *p* < 0.01.

It has been demonstrated that focal cerebral ischemia leads to a ceramide accumulation in the ischemic cerebral cortex [42]. Therefore, we studied the alterations after murine focal ischemia in the spleen and ischemic cerebral cortex [42]. In the spleen (Figure 6A), sphingosine (Sph), the precursor of S1P, was markedly reduced 24 h after MCAO, although statistic testing was not possible since Sph levels in sham mice were consistently above the upper limit of quantification (ULOQ) (sham vs. MCAO; ULOQ vs. 1644 ± 196.9 pg/mg, Figure 6A). Besides, we could not establish any significant difference in sphinganine (Sgn) levels 24 h after MCAO compared to sham-treated mice (sham vs. MCAO; 473.5 ± 221.7 pg/mg vs. 436.9 ± 78.7 pg/mg, *p* > 0.9999, Figure 6A). C16 concentration in murine spleen showed a striking increase 24 h post-occlusion (sham vs. MCAO; 1506 ± 646.8 pg/mg vs. 3637 ± 361.6 pg/mg, *p* = 0.0095, Figure 6A). Moreover, we demonstrated a significant increase in C18 ceramide levels as well compared to sham-operated mice (sham vs. MCAO; 318.8 ± 54.1 pg/mg vs. 770.2 ± 163.2 pg/mg, *p* = 0.004, Figure 6A). C20 and C24 ceramide levels showed a similar pattern (C20: sham vs. MCAO; 696.6 ± 138.6 pg/mg vs. 1323 ± 293.4 pg/mg, *p* = 0.004; C24: sham vs. MCAO; 2659 ± 843.3 pg/mg vs. 4900 ± 836.1 pg/mg, *p* = 0.0081, Figure 6A).

In contrast to the spleen, all ceramide species tested showed no difference in the brain as a consequence of MCAO (Sph: sham vs. MCAO; 788.3 ± 194.1 pg/mg vs. 900.0 ± 331.7 pg/mg, *p* = 0.2853; Sgn: sham vs. MCAO; 170.0 ± 60.7 pg/mg vs. 188.9 ± 74.2 pg/mg, *p* = 0.6885; C16 ceramide: sham vs. MCAO; 2735 ± 964.0 pg/mg vs. 2497 ± 1021 pg/mg, *p* = 0.607; C18 ceramide: sham vs. MCAO; 107.5 ± 21.8 ng/mg vs. 124.8 ± 37.2 ng/mg, *p* = 0.4559; C20 ceramide: sham vs. MCAO; 3553 ± 575.0 pg/mg vs. 3674 ± 1283 pg/mg, *p* = 0.8639; C24 ceramide: sham vs. MCAO; 3248 ± 2184 pg/mg vs. 4483 ± 1808 pg/mg, *p* = 0.2721, Figure 6B).

## 3. Discussion

This study provides comprehensive data on S1P concentrations in the murine brain, in the circulation, and in secondary lymphoid organs after MCAO and their association with systemic adaptations of the immune system. We found a steep S1P gradient with lowest concentrations in the spleen, moderate concentrations in the circulation, and highest concentrations in the ischemic core 24 h after MCAO. High S1P concentrations in the brain persisted directly in the ischemic lesion 24 h after MCAO with an additional gradient formed between the ischemic core (S1P^HIGH^) and the peri-infarct cortex (S1P^LOW^). We found the S1P gradient to be linked to splenic S1P_1_^+^ T cell egress. These evading T cells, being of T_H_ and T_REG_ cell phenotype, were swiftly recruited towards the brain almost instantly after MCAO. Unaltered ceramide levels but a differential expression of S1P_R_ were observed in the brain after stroke. We suggest that a differential expression of S1P_R_ may lure T_H_ and T_REG_ cells towards an S1P gradient and may be involved with the spatiotemporal positioning of these cells after acute ischemic stroke.

S1P signaling is conceived to be involved in regulating immune responses [26]. Erythrocytes and vascular endothelial cells have been identified as the key determinants of plasma S1P levels [43,44]. The S1P concentration is influenced by the actions of sphingosine kinase 1 (SphK1) and sphingosine kinase 2 (SphK2) [45]. The S1P levels in the blood and lymph are higher compared to tissues [46]. In the circulation, bound to ApoM^+^-HDL and albumin, S1P reaches high nanomolar concentrations. In contrast, low nanomolar concentrations are observed intracellularly and in interstitial fluids [47]. It was postulated that the chemotactic S1P gradient plays a role in lymphocyte egress directing lymphocytes into the circulation [4,13,48]. Accordingly, disrupting the S1P gradient by 2-acetyl-4-tetrahydroxybutylimidazole, an inhibitor of the S1P lyase, led to lymphopenia and inhibited T cell egress from the thymus, which was mediated by a downregulation of surface S1P_1_ [49]. Moreover, a deficiency in S1P_1_ expression was found to impair lymphocyte egress from secondary lymphoid organs along the S1P gradient [50]. Pharmacological manipulation of S1P_1_ through fingolimod led to T and B cell sequestration through internalization of S1P_1_ suggesting the S1P-S1P_1_ interaction to be the molecular switch of lymphocyte egress [4]. Sustained exposure to S1P downregulates S1P_1_ resulting in non-responsiveness to S1P-mediated chemotaxis and vice versa [51]. As it appears, the key enzymes SphK1/2 are inversely regulated when S1P_1_ is ligated by S1P on naïve T cells in the circulation [43]. Furthermore, these lymphocytes failed to egress from the thymus and secondary lymphoid organs [43].

We observed a steep S1P gradient towards the ischemic core after MCAO, whilst S1P was reduced in the peri-infarct cortex. Our findings align well with a study by Hasegawa et al. [19] who demonstrated that S1P_1_, SphK1, and SphK2 were decreased in the infarct cortex but preserved in the peri-infarct cortex at least 6 h after MCAO. With decreased levels of S1P in the penumbra, the negative feedback loop of S1P ligation to S1P_1_ is inhibited, resulting in maintained expression of S1P_1_ and active SphK1/2, respectively [43].

It was shown that NK cell infiltration into the ischemic hemisphere is an extremely swift process happening as early as 3 h post injury [52]. Reports are accumulating that this process applies to adaptive lymphocytes, too. In that respect, various T_H_ cell populations and T_REG_ cells in the ischemic hemisphere have been found to amass on the first day after MCAO [53,54,55]. Moreover, it can be argued that neurons are an important source of S1P [56], suggesting that as cerebral blood flow (CBF) decreases and neuronal damage worsens, the release of S1P into the ischemic core could contribute to the maintained high S1P levels [40,57]. This, in turn, would establish a new S1P gradient allowing various immune cell populations to invade the peri-infarct cortex. Our data obtained by qPCR and quantitative immunohistochemistry support the hypothesis of T_H_ and T_REG_ cell recruitment to the ipsilateral hemisphere after MCAO.

How these cells respond chemotactically to S1P may be subject to their unique S1P_R_ repertoire [39,58], which we found to be differentially patterned after MCAO. Interestingly, the S1P_R_ expression pattern was also altered in the contralateral hemisphere after stroke suggesting an additional regulatory function. It has been shown that the S1P_2_ is more abundantly expressed on vascular endothelial cells after cerebral ischemia [17], involved in vascular permeability and inflammation [59,60] with potential influence on lymphocyte traffic to and within the brain [61]. Moreover, S1P_2_ was recently linked to a pro-inflammatory response by inducing M1 polarization after cerebral ischemia [62]. In contrast, S1P_3_ is expressed in embryonic endothelial cells and is required for endothelial cell morphogenesis as well as migration [63,64,65]. In addition, S1P_3_ acts as a mediator of P-selectin-mobilizing effects by activating SphK1 [66].

In our study, we report a profound increase in the mRNAs for S1P_2_ and S1P_3_ correlating with enhanced CD3 and FoxP3 expression (using qPCR and IHC) 24 h after MCAO predominantly in the ipsilateral hemisphere. Studies have described the unique role of S1P_3_ in promoting inflammatory responses, mediated through its upregulation in astrocytes [67,68,69], the activation of RhoA and induction of COX-2, IL-6, and VEGF-α [70]. S1P_3_ has also been described to recruit macrophages to the site of inflammation [71]. We have found an upregulation of S1P_3_ in the ischemic brain in accordance with a recent report [40].

In our study, we report higher S1P levels in murine and human plasma compared to secondary lymphoid organs. This may allow lymphocyte egress from the spleen into the circulation. Nevertheless, lymphocytes in the circulation swiftly decreased 3 h after MCAO. In accordance with this finding, previous reports have shown that migration of immune cells is a process which almost coincides with MCAO [53,54,55]. However, the S1P gradient alone cannot solely account for immune cell migration to the brain as also sham S1P levels in the brain superseded sham S1P levels in the circulation. This suggests that other events such as the presentation of E-selectin by activated endothelium [72], or the expression of L-selectin by activated white blood cells [73,74] may be required for lymphocyte diapedesis in S1P_2_-loosened endothelial tightness adjacent to the peri-infarct cortex. Additionally, we suggest that the expression of S1P_1–5_ or other glycosphingolipids such as ceramides may foster/mediate immune cell chemotaxis to the brain. In that respect, we report a significant reduction in splenic S1P_1_ mRNA, in accordance with an early activation and egress of splenocytes [36]. In this study it was shown that a rapid splenic T cell response was established after the insult had occurred [36]. Moreover, tonsil-resident T cells were reported to be promptly recruited chemotactically into the circulation in response to S1P [48]. However, a contrasting effect was observed towards S1P as soon as these T cells had been recruited into the circulation and this effect was attributed to an altered ratio between S1P_1_ and S1P_2_ [48]. We hypothesize that the balanced expression of S1P_R_ may be subject to change upon activation and recruitment to the effector cell pool.

With regard to ceramides, their complex effects on immune cells have become more and more unraveled. They have been shown to induce apoptosis [75], and to be involved in a re-assembly of the T cell cytoskeleton by intercalating into the plasma membrane due to their lipophilic properties [76]. Here, we showed no significant changes in the level of ceramides in the peri-infarct cortex compared to sham. In contrast, we report increased ceramide levels in the spleen after MCAO, most likely due to the incitement of apoptosis.

Our findings regarding the S1P receptor expression profile after stroke in different compartments indicate an involvement of the systemic and local S1P signaling in immune cell trafficking after stroke. Contributing events, such as inflammation at the stroke site, allowing E-selectin to be expressed by the inflamed endothelium, S1P_2_ to reduce the extent of tight junctions established by S1P_1_, and L-selectin expression on activated lymphocytes may all contribute towards T_H_ and T_REG_ cell rolling and diapedesis through vessels traversing cerebral areas possessing S1P**^HIGH^** concentrations.

Taken together, our findings suggest relevant signaling effects triggered by S1P essentially regulating T_H_ and T_REG_ cell responses, a differential patterning of S1P_R_ and their swift recruitment towards the injured site and renders ceramides unlikely to regulate lymphocyte recruitment to the brain.

## 4. Conclusions

We found a steep S1P gradient towards the brain after MCAO in mice and corroborated the clinical significance with a parallel significant increase of plasma S1P after stroke. This was accompanied by a drop of circulating lymphocytes in the context of lower S1P levels in the spleen. Our findings of intricate and organ-specific regulations of the S1P receptor expression profile after acute ischemic stroke suggest systemic and local S1P signaling to be involved in regulating the lymphocyte recruitment towards the peri-infarct region of the brain and systemic stroke-induced immunosuppression. Future studies remain to elucidate the causal effect that S1P_R_ patterns impose on spatiotemporal positioning and chemotaxis of T_H_ and T_REG_ cells within the brain. Understanding how these cells can be therapeutically utilized to reduce the detrimental effects caused by ischemic stroke may help to identify pharmacologically exploitable targets for an improved recovery and outcome in patients suffering from ischemic stroke.

## 5. Materials and Methods

For all experiments, male C57BL/6 mice (strain J, 11–12 weeks, Charles River Laboratories, Sulzfeld, Germany) were used and kept on a 12:12 h light-dark cycle with food and water ad libitum. All animal experiments in this study conformed to the German Protection of Animals Act and the guidelines for care and use of laboratory animals by the local committee (Regierungspräsidium Darmstadt, Germany, FU/1049, 2 April 2015).

### 5.1. Experimental Model of Middle Cerebral Artery Occlusion

Transient right MCAO was performed for 1 h under anesthesia with 1.5% isoflurane (Abbott, Wiesbaden, Germany) and 0.1 mg/kg buprenorphine (Essex Pharma, Munich, Germany) under spontaneous respiration using a standardized silicon-coated monofilament with a tip diameter of 0.23 mm (Doccol, Redlands, CA, USA). A midline cervical incision was performed, and the right carotid bifurcation was exposed. The monofilament was introduced and advanced along the internal carotid artery occluding the proximal stem of the middle cerebral artery. The reperfusion was initiated by withdrawing the filament after 1 h of focal cerebral ischemia. Following the operation, mice were monitored until regaining consciousness and returned to their cages. All animals received food and regular drinking water ad libitum. Animals were assessed either 3 or 24 h after reperfusion.

In total, 32 mice were subjected to 1 h of MCAO and harvested 24 h post-intervention, 10 mice were harvested after 3 h whilst, 15 sham-operated mice were harvested after 24 h and employed as controls. Death as a result of the MCAO operation was the sole exclusion criterion in this study. In total, three mice had died (mortality rate: 3/57 = 5.3%) and were therefore excluded from further experimental investigation. All other animals that were subjected to MCAO displayed clear features of ischemia as analyzed by the mNSS score and TTC staining, respectively. The operations were performed unblinded since the operator did not apply any modifications such as drug treatment. However, sample provision for flow cytometry, immunohistochemistry, mass spectrometry, and qPCR assessment were done in a blinded fashion.

### 5.2. Determination of Sphingolipid Concentrations by High-Performance Liquid Chromatography-Tandem Mass Spectrometry

The quantification of sphingolipids was performed for brain or serum. In total, 10 μL serum was used for lipid analysis. Serum samples were mixed with 190 μL water before the extraction. The samples were mixed with 200 μL extraction buffer (citric acid 30 mM, disodium hydrogen phosphate 40 mM) and 20 μL of the internal standard solution containing sphingosine-d7, sphinganine-d7 (200 ng/mL each), and C18-sphingosine-1-phosphate-d7 (400 ng/mL methanol, all Matreya, State College, PA, USA). The mixture was extracted once with 1000 μL methanol:chloroform:hydrochloric acid (15:83:2, *v/v/v*). The organic phase was evaporated and reconstituted in 100 μL of tetrahydrofuran:water (9:1, *v/v*) containing 0.2% formic acid and 10 mM ammonium formate. 

For the determination of the tissue samples, the samples were mixed with 200 µL water and 20 µL internal standard solution and homogenized using a Mixer Mill MM400 (Retsch, Haan, Germany) with five zirconium oxide grinding balls for each sample (25 Hz for 2.5 min). The extraction was processed as described for serum but using 10 µL of the homogenate. For calibration standards and quality control samples preparation, 20 µL of the corresponding working solutions were processed as stated instead of sample.

C20-sphingosine-1-phosphate was determined semiquantitatively by means of a C20-S1P standard. An Agilent 1100 series binary pump (Agilent Technologies, Waldbronn, Germany) equipped with a Luna C8 column (150 × 2 mm ID, 3 μm particle size, 100 Å pore size; Phenomenex, Aschaffenburg, Germany) was used for chromatographic separation under gradient conditions. The HPLC mobile phases consisted of water with 0.2% formic acid and 2 mM ammonium formate (mobile phase A) and acetonitrile:isopropanol:acetone (50:30:20, *v/v/v*) with 0.2% formic acid (mobile phase B). The total running time was 21 min and the injection volume was 15 μL. Acetonitrile with 0.1% formic acid was infused post-column using an isocratic pump at a flow rate of 0.15 mL/min. The MS/MS analyses were performed using a triple quadrupole mass spectrometer API4000 (Sciex, Darmstadt, Germany) equipped with a Turbo V Ion Source operating in positive electrospray ionization Multiple Reaction Monitoring mode. 

Data Acquisition was done using Analyst Software V 1.6 and quantification was performed with MultiQuant Software V 3.0 (both Sciex), employing the internal standard method (isotope dilution mass spectrometry).

### 5.3. Flow Cytometry Analysis of Immune Cells

Brain, blood, and spleen tissues were collected for flow cytometric analyses. Tissue samples were harvested, and single-cell suspensions were obtained. Blood was acquired through cardiac puncture from the right ventricle. Each blood sample, approximately 500 μL blood, was drawn with a syringe filled with heparin solution to prevent clotting and transferred to a Falcon tube with ice-cold 15 mL RPMI-1640 cell culture medium. Falcon tubes were centrifuged (5 min, 1000 rpm, 4 °C) and the supernatant was discarded. Cells were incubated with blocking buffer (100 µL PBS + 1% FCS + 0.01%NaN_3_) and subsequently stained with fluorochrome-conjugated antibodies for 30 min in the dark at 4 °C. Samples were analyzed on a BD FACSCanto II (BD Biosciences, Heidelberg, Germany). The following antibodies were used: CD45-FITC (clone 30F11, Miltenyi Biotec, Bergisch Gladbach, Germany), CD45-PE-Cyanine 7 (Miltenyi Biotec, Bergisch Gladbach, Germany), CD3ε-APC-Vio770 (clone 145-2C11, BD Miltenyi Biotec, Bergisch Gladbach, Germany), CD19-VioBlue (clone 6D5, Miltenyi Biotec, Bergisch Gladbach, Germany), CD11b-APC (clone REA592, Miltenyi Biotec, Bergisch Gladbach, Germany), Ly6G-PE (clone REA526, Miltenyi Biotec, Bergisch Gladbach, Germany), Ly6C-FITC (clone 1G7.G10, Miltenyi Biotec, Bergisch Gladbach, Germany), and S1P_1_-eFluor660 (clone SW4GYPP, ThermoFisher, Darmstadt, Germany). With respect to the identification of various immune cells in murine compartments cell lineage ontogeny was considered as follows: CD45^+^ cell (commonly expressed by pan-leukocytes and pan-lymphocytes, i.e., differentiated hematopoietic cells), leukocytes (CD45^+^CD11b^+^), PMN (CD45^+^Ly6G^+^), monocytes (CD45^+^Ly6C^MODERATE^), dendritic cells (CD45^+^Ly6C^HIGH^), B cells (CD45^+^CD19^+^), T cells (CD45^+^CD3^+^), T_H_ cells (CD45^+^CD4^+^).

### 5.4. RNA Isolation, cDNA Synthesis, and Quantitative Real-Time PCR

Total mouse RNA from splenocytes and brain homogenates were extracted using Tri reagent (Sigma-Aldrich T9424, Taufkirchen, Germany) according to the manufacturer’s instructions. In brief, the spleen was incised and transferred to an Eppendorf tube containing 1 mL of RPMI medium containing GlutaMAX (Gibco, ThermoFisher Scientific, #61870-010, Darmstadt, Germany). The spleen was dissociated using 70 µM Falcon cell strainer (Corning, #352350) and cells were collected in a 50 mL Falcon tube. The cell suspension was centrifuged (400 rpm, 5 min), followed by red blood cell lysis. Then, 1 mL of Tri reagent was added to the cell pellet and frozen immediately. With respect to the brain, after removal from the skull, the ischemic and contralateral hemispheres were separated and transferred to 15 mL Falcon tubes containing 5 mL of PBS (Gibco, ThermoFisher Scientific, #14287-080, Darmstadt, Germany). Homogenization was achieved using the brain dissociation kit (#130-107-677, Miltenyi Biotec, Bergisch Gladbach, Germany). Again, 1 mL of Tri reagent was added to 100 µL of brain homogenate and RNA was isolated according manufacturer’s instructions. The RNA yield was established by NanoDrop (ThermoFisher, Darmstadt, Germany). The A260/A280 ratio at this point was routinely between 1.9 and 2.1. Three technical replicates were used for each condition. A total of 1200 ng of RNA was reverse transcribed into cDNA using the RevertAid First Strand cDNA Synthesis Kit (ThermoFisher Scientific, #K1621, Darmstadt, Germany). Using oligo dT primers, a TaqMan-based real-time PCR quantitation was performed (Applied Biosystems 7500fast, Darmstadt, Germany). Duplex PCR was performed using the following cycling parameters: 95 °C (2 min (only 1st cycle)); 95 °C (5 s) followed by 62 °C (30 s) for 40 cycles. Relative mRNA abundance was calculated using the comparative delta-delta Ct method. The Ct values were normalized by the target mRNA/GAPDH gene average value for all samples. The following TaqMan probes (ThermoFisher Scientific, Darmstadt, Germany) were used: GAPDH (Mm99999915), CD3d (Mm00442746), CD11b [ITGAM] (Mm00434455), IL-6 (Mm00446190), PU.1 [SPI1] (Mm00488140), STAT3 (Mm01219775), FoxP3 (Mm00475162), S1P_1_ (Mm02619656), S1P_2_ (PN4441114), S1P_3_ (Mm02620181), S1P_4_ (Mm00468695), and S1P_5_ (Mm02620565).

### 5.5. Immunohistochemistry

The 3 µm thick paraffin sections were subjected to deparaffinization/hydration as follows: (1) suspension in xylene for 10 min for four times; (2) 5 min in isopropanol twice; (3) 5 min in 96% ethanol twice; (4) 5 min in 70% ethanol; (5) 10 min in double distilled water twice. Antigen unmasking was performed by boiling slides in a citrate-based target retrieval solution (Agilent Dako #S1699, Jena, Germany) for 20 min. Then, the slides were cooled to room temperature and the sections were washed in PBS twice, followed by incubation in 0.1% Triton X-100-PBS for 4 min. The slides were washed with PBS two more times. The slides were blocked for 45 min using an immunoblock solution (Roth #T144.1, Karlsruhe, Germany). The primary antibody against CD3 (clone M-20, Santa Cruz Biotechnology, #sc-1127) or FoxP3 (clone 2A11G9, Santa Cruz Biotechnology, #sc-53876, Dallas, TX, USA) diluted 1:50 in the immunoblock solution was added to the slides and was allowed to incubate for ≥16 h at 4 °C. Signal detection was obtained using Histofine Simple Stain Max PO detection system (#414161F, Nichirei Biosciences Inc., Tokyo, Japan) and DAB peroxidase substrate kit (#SK-4100, Linaris, Vector laboratories, Dossenheim, Germany). Counterstaining was employed using hematoxylin. Finally, images were acquired on a Keyence microscope (BZ-8000K, Osaka, Japan).

### 5.6. Statistical Analyses

GraphPad Prism 8 (GraphPad Software, LLC, La Jolla, CA, USA) was used for statistical analyses. Results are expressed as median ± interquartile range (IQR) except stated otherwise. Statistical significance was assessed using Wilcoxon test for paired samples and Mann–Whitney or Kruskal–Wallis test for unpaired samples. A *p* value of <0.05 was considered statistically significant.

## Supplementary Materials

The following are available online at https://www.mdpi.com/1422-0067/21/17/6242/s1, Figure S1: Temporal profile of the murine spleen weight after MCAO, Figure S2: Temporal profile of immune cell egress from the murine spleen after MCAO, Figure S3: Splenic CD3+ T cell egress, Figure S4: Quantification of CD3+ T cell recruitment to the brain, Figure S5: Quantification of FoxP3+ TREG cell recruitment to the brain.

## Abbreviations

ApoM	Apolipoprotein M
CBF	Cerebral blood flow
CD	Cluster of differentiation molecule
CL	Contralateral
CNS	Central nervous system
COX-2	Cyclooxygenase-2
FC	Fold change
FoxP3	Forkhead box protein P3
GAPDH	Glyceraldehyde-3-phosphate dehydrogenase
IL	Ipsilateral
IL-6	Interleukin-6
IQR	Interquartile range
IR	Ischemia/reperfusion
MCAO	Middle cerebral artery occlusion
mRNA	Messenger ribonucleic acid
PIC	Peri-infarct cortex
PVA	Periventricular area
qPCR	Quantitative real-time polymerase chain reaction
RhoA	Transforming protein RhoA
SCID	Severe combined immunodeficiency
SphK1	Sphingosine kinase 1
SphK2	Sphingosine kinase 2
SPI1	Transcription factor PU.1
STAT3	Signal transducer and activator of transcription 3
S1P	Sphingosine 1-phosphate
S1P_1_	Sphingosine 1-phosphate receptor 1
S1P_2_	Sphingosine 1-phosphate receptor 2
S1P_3_	Sphingosine 1-phosphate receptor 3
S1P_4_	Sphingosine 1-phosphate receptor 4
S1P_5_	Sphingosine 1-phosphate receptor 5
S1PR	Sphingosine 1-phophate receptor
VEGF-α	Vascular endothelial growth factor A
