# Supplementary material for: A Sphingosine 1-Phosphate Gradient Is Linked to the Cerebral Recruitment of T Helper and Regulatory T Helper Cells during Acute Ischemic Stroke
Source: Int J Mol Sci. 2020 Aug 28;21(17):6242. doi: 10.3390/ijms21176242 (PMC7503682; doi:10.3390/ijms21176242)
Supplement: Supplementary file 1 [file ijms-21-06242-s001.pdf]

Article

A Sphingosine 1-Phosphate Gradient is Linked to the Cerebral Recruitment of T Helper and Regulatory T Helper Cells during Acute Ischemic Stroke

Alexandra Lucaciu [1,*], Hannah Kuhn [1], Sandra Trautmann [2], Nerea Ferreirós [2], Helmuth Steinmetz [1], Josef Pfeilschifter [3], Robert Brunkhorst [1,4], Waltraud Pfeilschifter [1], Julien Subburayalu [5,†] and Rajkumar Vutukuri [3,*,†]

[1] Department of Neurology, Goethe University Frankfurt, 60528 Frankfurt am Main, Germany; alexandra.lucaciu@kgu.de (A.L.); hannah.kuhn@gmail.com (H.K.); h.steinmetz@em.uni-frankfurt.de (H.S.); rbrunkhorst@ukaachen.de (R.B.); waltraud.pfeilschifter@kgu.de (W.P.)

[2] Institute of Clinical Pharmacology, Pharmazentrum Frankfurt, Goethe University Frankfurt, 60528 Frankfurt am Main, Germany; trautmann@med.uni-frankfurt.de (S.T.); ferreirosbouzas@em.uni-frankfurt.de (N.F.)

[3] Institute of General Pharmacology and Toxicology, Pharmazentrum Frankfurt, Goethe University Frankfurt, 60528 Frankfurt am Main, Germany; pfeilschifter@em.uni-frankfurt.de (J.P.); vutukuri@med.uni-frankfurt.de (R.V.)

[4] Department of Neurology, RWTH Aachen University, 52074 Aachen, Germany

[5] Department of Medicine, University of Cambridge, Cambridge CB2 0QQ, UK; js2380@cam.ac.uk (J.S.)

* Correspondence: alexandra.lucaciu@kgu.de (A.L.); vutukuri@med.uni-frankfurt.de (R.V.)

† These authors contributed equally.

Received: 07 July 2020; Revised: 12 August 2020; Accepted: 26 August 2020; Published: date

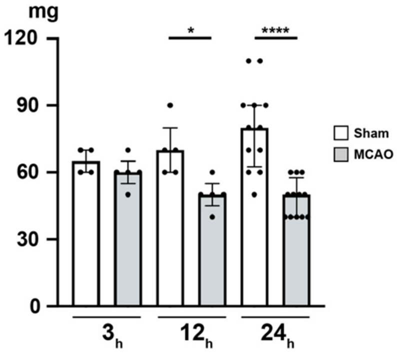

Figure S1. Temporal profile of the murine spleen weight after MCAO. The figure depicts the changes in splenic weight 3, 12, and 24 h after MCAO compared to sham-operated mice. The Mann–Whitney U-test was applied to calculate statistical differences. The data are presented as median ± IQR; $^{*}p < 0.05$, $^{****}p < 0.0001$.

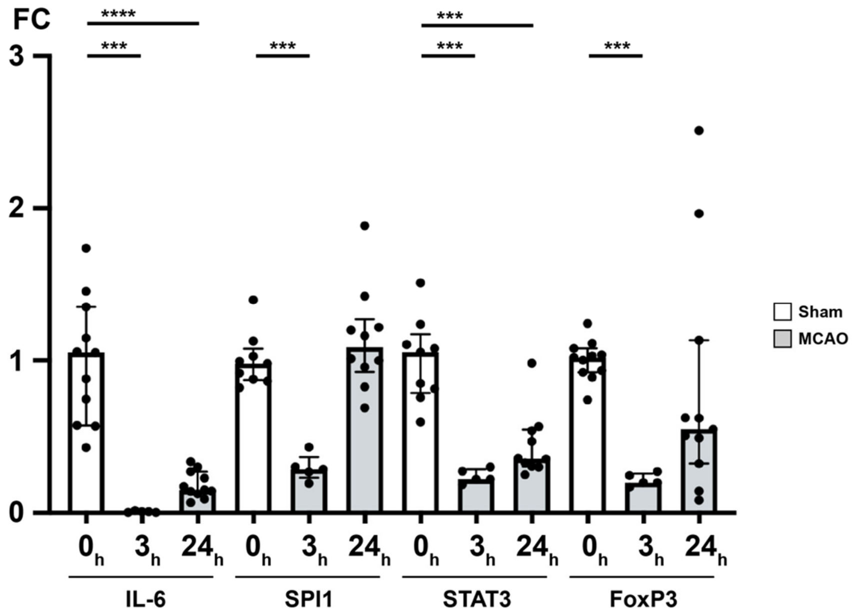

Figure S2. Temporal profile of immune cell egress from the murine spleen after MCAO. The figure shows the variations in the fold change (FC) of IL-6 and the transcription factors SPI1, STAT3, and FoxP3 in the murine spleen (3 and 24 h) after MCAO compared to sham. Fold change (FC) was normalized to the expression of GAPDH mRNA levels. The Mann–Whitney U-test was applied to calculate statistical differences. Data are presented as median ± IQR; **p* < 0.05, ***p* < 0.01.

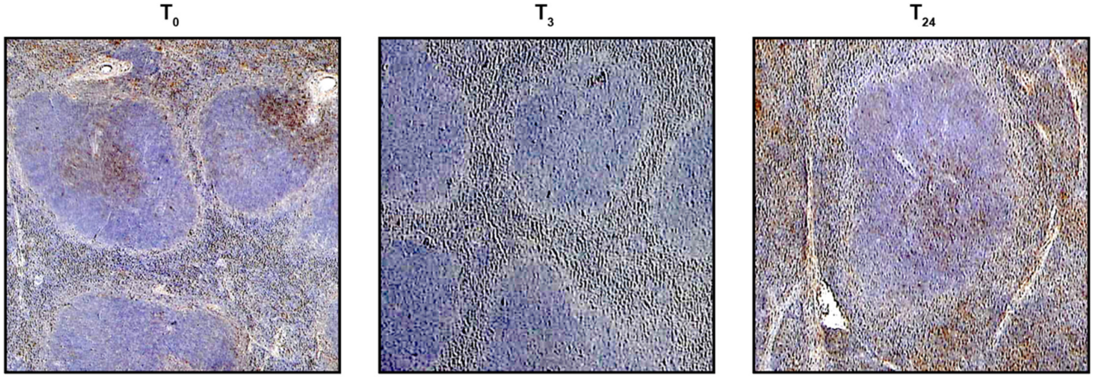

Figure S3. Splenic $CD3^+$ T cell egress. Temporal CD3 immunohistochemistry of sections from MCAO-operated mice. CD3 immunostaining is swiftly lost both from the white pulp, but equally, from the associated red pulp 3 h post-intervention (T_3). CD3 expression is regained in later stages after ischemia, suggesting a re-pooling of T cells back to the spleen.

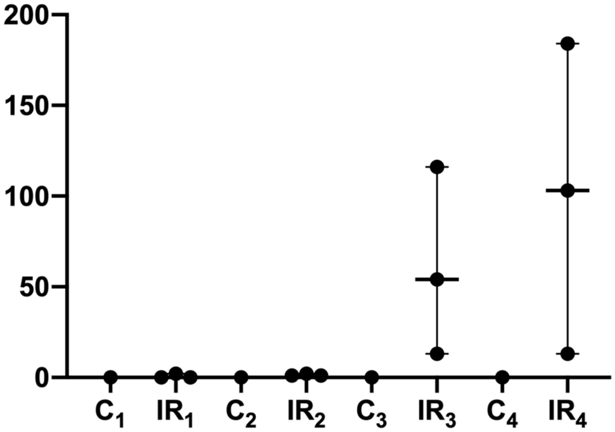

Figure S4. Quantification of $CD3^+$ T cell recruitment to the brain. The figure details the number of infiltrated $CD3^+$ T cells into the various cerebral areas denoted in Figure 4, i.e., region 1 (contralateral PIC), region 2 (contralateral peri-ventricular area (PVA)), region 3 (ipsilateral PVA), and region 4 (ipsilateral PIC). For each region, a comparison is made between sham-operated (C) and MCAO-operated mice (IR). Data are presented as median ± IQR; $n = 3$.

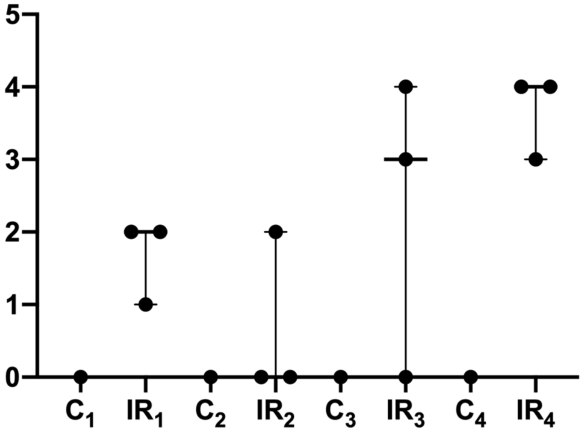

Figure S5. Quantification of $FoxP3^+$ Treg cell recruitment to the brain. The figure details the number of infiltrated $FoxP3^+$ T cells into the various cerebral areas denoted in Figure 5, i.e., region 1 (contralateral PIC), region 2 (contralateral peri-ventricular area (PVA)), region 3 (ipsilateral PVA), and region 4 (ipsilateral PIC). For each region, a comparison is made between sham-operated (C) and MCAO-operated mice (IR). Data are presented as median ± IQR; $n = 3$.
